# Research on Online Nondestructive Detection Technology of Duck Egg Origin Based on Visible/Near-Infrared Spectroscopy

**DOI:** 10.3390/foods12091900

**Published:** 2023-05-06

**Authors:** Qingxu Li, Wanhuai Zhou, Qiaohua Wang, Dandan Fu

**Affiliations:** 1 Department of Computer Science, Anhui University of Finance and Economics, Bengbu 233030, China; 120220059@aufe.edu.cn (Q.L.); 120140003@aufe.edu.cn (W.Z.); 2 College of Engineering, Huazhong Agricultural University, Wuhan 430070, China; 3 College of Mechanical Engineering, Wuhan Polytechnic University, Wuhan 430023, China; fddwhpu@gmail.com

**Keywords:** duck eggs, origin, visible/near-infrared, online detection, device

## Abstract

As living standards rise, people have higher requirements for the quality of duck eggs. The quality of duck eggs is related to their origin. Thus, the origin traceability and identification of duck eggs are crucial for protecting the rights and interests of consumers and preserving food safety. As the world’s largest producer and consumer of duck eggs, China’s duck egg market suffers from a severe lack of duck egg traceability and rapid origin identification technology. As a result, a large number of duck eggs from other regions are sold as products from well-known brands, which seriously undermines the rights and interests of consumers and is not conducive to the sound development of the duck egg industry. To address the above issues, this study collected visible/near-infrared spectral data online from duck eggs of three distinct origins. To reduce noise in the spectral data, various pre-processing algorithms, including MSC, SNV, and SG, were employed to process the spectral data of duck eggs in the range of 400–1100 nm. Meanwhile, CARS and SPA were used to select feature variables that reflect the origin of duck eggs. Finally, classification models of duck egg origin were developed based on RF, SVM, and CNN, achieving the highest accuracy of 97.47%, 98.73%, and 100.00%, respectively. To promote the technology’s implementation in the duck egg industry, an online sorting device was built for duck eggs, which mainly consists of a mechanical drive device, spectral software, and a control system. The online detection performance of the machine was validated using 90 duck eggs, and the final detection accuracy of the RF, SVM, and CNN models was 90%, 91.11%, and 94.44%, with a detection speed of 0.1 s, 0.3 s, and 0.5 s, respectively. These results indicate that visible/near-infrared spectroscopy can be exploited to realize rapid online detection of the origin of duck eggs, and the methodologies used in this study can be immediately implemented in production practice.

## 1. Introduction

Duck eggs are rich in protein and trace elements [1]. Hence, duck eggs are particularly popular among Chinese consumers. According to a report by the China Animal Farm Association and the Food and Agricultural Organization of the United Nations (FAO), China’s duck egg production reached 2.776 million tons in 2021. In China’s duck egg market, a large number of duck eggs from other regions are sold with well-known brands such as Gaoyou and Baiyangdian. This severely undermines the rights of customers who are paying higher prices for non-branded goods. The duck breeds raised in different regions of China vary substantially, and climatic circumstances in various regions differ greatly, resulting in various approaches to duck farming. This leads to a significant disparity in the quality of duck eggs from different regions [2,3]. Thus, the origin identification and traceability of duck eggs is one of the most effective means of addressing the confusion in the Chinese duck egg market, which is crucial for protecting consumer rights and ensuring food safety.

Researchers have employed stable isotope techniques [4], hyperspectral techniques [5], and nuclear magnetic resonance to determine the traceability and origin of poultry eggs [6]. Although the stable isotope approach has a high level of detection accuracy, it is time-consuming, difficult to operate, and needs to break eggs. Although hyperspectral technology can collect both image and spectral data of eggs, its high equipment cost makes it unsuitable for industrial use. Nuclear magnetic resonance technology has similar disadvantages to the two preceding technologies, i.e., it is expensive and difficult to operate. To address the above issues, Liu et al. employed FT-NIR to detect the origin of fresh chicken eggs [7]. The origin identification and traceability of duck eggs are crucial, but there is no research on this, especially without destroying the eggshell. Meanwhile, compared to chicken eggs, it is more difficult to detect and track the origin of duck eggs, mostly due to the thicker eggshell and dirtier surface of duck eggs.

Visible/near-infrared spectroscopy consists of the combined and multiplied frequencies of the vibrations of hydrogen-containing groups of organic substances, and the spectrum contains a large amount of information that can reflect the differences between substances. It is quick, nondestructive, and simple, and is now widely used in the nondestructive testing of eggs [8,9]. For instance, Puertas et al. used this technique to measure the cholesterol content of eggs [10], while Yuan et al. used it to determine the Haugh Unit, a measure of egg quality [11]. Aboonajmi et al. investigated the use of visible/near-infrared spectroscopy to assess freshness [12], Abdel-Nour et al. for protein content [13], and Dong et al. for fertilization status [14]. These studies demonstrate the advantages of visible/near-infrared spectroscopy in detecting internal substances in eggs. As Liu et al. note, differences between eggs of different origins are mainly due to internal substances [7]. Thus, visible/near-infrared spectroscopy could be a viable method for detecting the origin of duck eggs. Additionally, convolutional neural networks (CNN) have advantages in dealing with classification problems and are commonly used in the detection of agricultural and food products [15,16]. Therefore, researchers employ CNN in combination with spectral approaches to classify softwood species [17], tobacco leaves [18], and coal [19].

Visible/near-infrared spectroscopy has been demonstrated to penetrate the eggshell and detect the interior substances of eggs [20]. The differences between eggs of various origins and farming methods are reflected in the interior components of the eggs [21]. Consequently, this paper proposes to construct an online collection system for visible/near-infrared transmission spectral information of duck eggs from three distinct origins. The online collection system consists of a spectrum data acquisition system and an automatic transmission device. After pre-processing the spectral data and extracting the feature wavelengths of duck eggs, this paper proposes to establish a duck egg origin classification model with CNN and deploy the classification model in the machine for online origin identification of duck eggs.

## 2. Methods and Materials

### 2.1. Sample Preparation

With a similar size and shape, and originating from the provinces of Jiangsu, Sichuan, and Henan, duck eggs of three distinct origins were chosen in this study. All the duck eggs from the same area originate from three distinct farms. The total number of duck eggs was 351, with 130 coming from Jiangsu, 110 from Sichuan, and 111 from Henan. Additionally, 261 duck eggs were utilized to develop the detection model (182 in the training set and 79 in the test set), and 90 were used to validate the performance of the online detection technique (Table 1). Before collecting spectral data from a duck egg, large stains on the surface of the egg were wiped away with 75% alcohol.

### 2.2. Online Spectral Data Acquisition System

**Figure 1:**
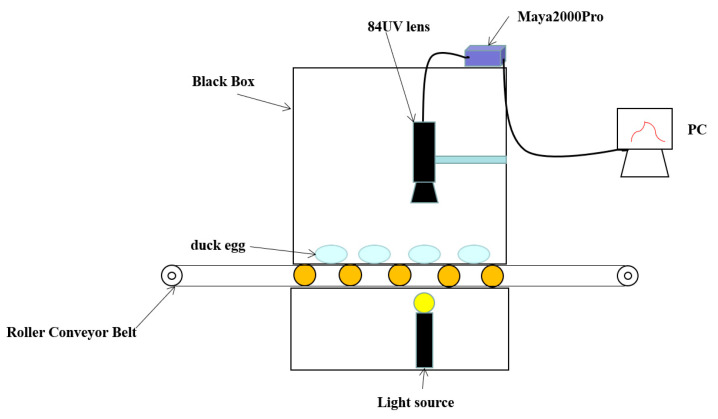
The spectral data acquisition system.

The spectral data acquisition system consisted of a computer, a Maya2000Pro spectrometer, a black box, a light source, an 84UV lens, and a roller conveyor belt (Figure 1). The Maya2000Pro spectrometer has a wavelength range of 200~1100 nm and a sample interval of 0.5 nm, together with an 84UV lens for collecting spectral data on duck eggs. The light source was a 100-watt tungsten halogen lamp with a wavelength range of 400~2500 nm, and it can penetrate the eggshell of a duck egg. The black box was made of metal, and its inside was sprayed with black paint. It can prevent external light from interfering with spectral data. The duck eggs were transported on the roller conveyor belt; while collecting spectral data, the eggs were placed horizontally so that they were in line with the light source and 84UV lens.

With the light source on, duck eggs were transported above by the motor-driven roller conveyor belt. Being connected to the 84UV lens and the Maya2000Pro spectrometer, the computer collected and saved the spectral duck egg data. The software for spectral data collection was developed and designed independently. The distance between the 84UV lens and the duck egg was 5 cm, and the duck egg was positioned horizontally. The software captured the duck egg’s spectral data in the form of a transmission spectrum. 

### 2.3. Spectral Data Processing and Modeling Methods

#### 2.3.1. Spectral Wavelength Selection

As the wavelength range of the light source and the spectrometer was 400~2500 nm and 200~1100 nm, respectively, the valid wavelength range for the duck egg’s spectral data was 400~1100 nm. This study chose spectral data between 400 and 1100 nm for duck egg origin detection and traceability.

#### 2.3.2. Pre-Processing of Spectral Data

There are numerous stains and rough surfaces on the eggshell of duck eggs, which can cause light scattering. Meanwhile, when collecting spectral data from duck eggs, the temperature change of the environment and the placement of the eggs may induce small movements of the yolk, both of which are random factors. The MSC algorithm can be adopted to reduce the noise caused by scattering [22], the SG algorithm can eliminate the effect of random noise on the spectral data and improve the signal-to-noise ratio of the spectral data [23], and the SNV algorithm can reduce the noise caused by scattering due to the size of solid particles and the uneven distribution of particles [24]. In this study, the MSC, SG, and SNV algorithms were used to reduce scattering and random noise in the spectral data of duck eggs.

#### 2.3.3. Feature Selection for Spectral Data

Feature selection is typically required prior to modeling with spectral data [25]. The spectral data of the duck eggs contain information that can be exploited to identify the origin of eggs, as well as a large amount of irrelevant data. Retaining the spectral information that can be used to identify the origin of duck eggs and deleting the unnecessary information can improve the model’s detection speed and performance [26]. The successive projections algorithm (SPA) and the competitive adaptive reweighted sampling (CARS) algorithm were used in this study to extract spectral data features that can reflect duck egg origin information. 

The SPA algorithm is a forward-loop-selection method that can minimize vector space collinearity [27]. The implementation of the SPA algorithm requires the specification of two parameters (the initial wavelength and the number of wavelengths to be extracted):

(a) Before the initial iteration, let $x_{j}$ represent the jth column of the spectrum of the training set, where j=1,2,…J, and J represent the total number of wavelengths.

(b) The non-selected columns are marked as set S.

(c) Calculate the projection vector of $x_{j}$ on $x_{k(n-1)}$ space, where $P_{x_{j}}$ is the projection operator and $x_{k(n-1)}$ is the vector of remaining wavelength points after the initial wavelength points have been removed.

(1) $$P_{x_{j}}=x_{j}-(x_{j}{}^{T}x_{k(n-1)})x_{k(n-1)}{\left(x_{k(n-1)}{}^{T}x_{k(n-1)}\right)}^{-1}$$

(d) The position of the largest projection vector that could be selected in the previous step is taken as the initial value for the next step, and the set with the smallest cross-validation mean squared error value is selected by constructing a multivariable correction model.

The CARS algorithm was adopted to select the wavelength points with relatively large absolute values of regression coefficients in the partial least squares model. Specifically, adaptive reweighted sampling means were employed, the wavelength points with relatively small weights were eliminated, and cross-validation was used to select the subset with the lowest cross-validation mean squared deviation values, which can effectively identify the optimal combination of variables [28]. The implementation procedure is as follows:

(a) Monte Carlo sampling. To construct a partial least squares model, a certain percentage of samples are selected at random from the data set. The spectral matrix to be measured is $X_{a\times b}$, where a is the number of samples, b is the number of variables, and $Y_{a\times b}$ is the component matrix. The equation of the partial least squares model is as follows:

(2) $$Y_{a\times 1}=X_{a\times b}k+E$$

The regression coefficient, the bias, and the weight coefficient are denoted by k, E, and $w_{i}$, respectively. $w_{i}$ is defined to measure the importance of each wavelength point, and wavelength points having a weight coefficient of 0 are eliminated.

(3) $$w_{i}=\frac{\left|k_{i}\right|}{\sum_{i=1}^{b}\left|k_{i}\right|}\;i=1,2,\ldots ,b$$

(b) After several rounds of Monte Carlo samplings, the residual rates of the wavelength variables are calculated, and then the variables are filtered by the weight coefficients to determine the optimal combination of variables when the cross-validation mean squared deviation is minimal.

#### 2.3.4. Modeling Methods

Random forest (RF), support vector machines (SVM), and CNN were employed to establish the classification model. The spectral data of duck eggs from Sichuan, Jiangsu, and Henan were labeled as “0”, “1”, and “2”, respectively. The establishment of the classification model includes training and validation.

RF consists of several decision trees, each of which is independent of the others. Each decision tree classifies samples and then uses the voting mechanism to complete the classification task. It performs well in classification and can handle high-dimensional data more effectively. The implementation process is as follows:

(a) Set N to the number of samples in the training set. Resume the sampling procedure multiple times to obtain the training set for the decision tree, where M is the number of features.

(b) Set m to the number of input features, and m must be much smaller than M, which is used to establish the optimal splitting point for each decision tree node.

(c) The decision tree finishes growing, no pruning operation is performed, and the training of the model is finally completed.

SVM is proposed based on statistical theory and structural risk minimization, and it can solve classification, regression, and distribution estimation problems. Its basic principle is to map the nonlinear problem into a high-dimensional feature space by selecting appropriate kernel functions and penalty factors and then constructing an ideal classification hyperplane for classification. The decision function of the model is shown below [29]:

(4) $$f(x)=sign(\sum_{i=1}^{N}a_{i}{}^{*}\exp(\frac{\left||x-z|\right|}{2\sigma^{2}})+b^{*})$$

where *x* denotes the feature vector, $a_{i}{}^{*}$ denotes the optimal solution, and $b^{*}$ denotes the optimal hyperplane.

CNN introduces the mechanism of local connectivity and weight sharing, which enables it to contain more hidden layers. Attributed to this, CNN has a distinct advantage in handling classification problems [30]. However, CNN requires a large number of training and test samples; thus, this study reviewed the research on the application of CNN to spectroscopy. Yu et al. employed a one-dimensional convolutional neural network (1D-CNN) using 120 samples to predict the pesticide residues in Hami melon [31]. Tian et al. used a 1D-CNN in combination with visible/near-infrared spectroscopy to detect freezing damage in oranges with 114 samples [32]. Bai et al. estimated soil organic carbon using CNN and visible/near-infrared spectroscopy, and 330 samples were used [33]. In our study, 261 duck eggs were used to construct the origin detection model. As long as the built CNN does not have a complex structure, this number of samples is sufficient.

After feature extraction by CARS or SPA, the spectral data of duck eggs is one-dimensional and not suitable for direct input to the CNN; therefore, the one-dimensional matrix must be converted into a two-dimensional matrix, and the conversion equation is as follows:

(5) $$S=X^{T}X$$

where X denotes one-dimensional spectral data, and $X^{T}$ is the transpose of one-dimensional spectral data. The two-dimensional spectral matrix contains the original information of the one-dimensional spectral data, reflecting the variance and covariance of the samples while adapting to the CNN’s input structure.

**Figure 2:**
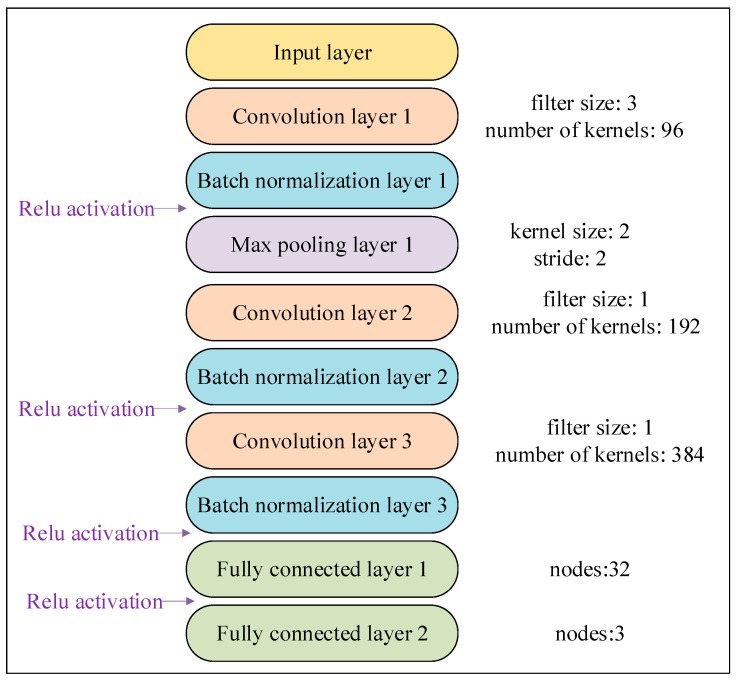
The CNN structure for the spectrum of duck eggs.

In this study, the small size of the two-dimensional spectral matrix of the duck egg is unsuitable for constructing a complex CNN. After numerous efforts, a CNN with 3 convolutional layers, 3 batch normalization layers, 2 fully connected layers, and 1 pooling layer was constructed (Figure 2). The network’s specific structure is as follows:

(a) Input layer: it transforms the duck egg spectral data processed by the SPA or CARS algorithm into a two-dimensional matrix.

(b) Convolution layer 1: the data of the input layer are subjected to the two-dimensional convolution operation with a 3 × 3 convolution filter and 96 convolution kernels.

(c) Batch normalization layer 1: Performing batch normalization on the data in the convolution layer 1 can prevent model overfitting, and activating the data using the ReLU function after this step can also prevent model overfitting. 

(d) Max pooling layer 1: it can reduce the data dimensionality of batch normalization layer 1 and the CNN’s computational complexity. The kernel size is 2, and the stride is 2.

(e) Convolution layer 2: the data of max pooling layer 1 are subjected to the two-dimensional convolution operation with a 1 × 1 convolution filter and 192 convolution kernels.

(f) Batch normalization layer 2: perform batch normalization on the data in convolution layer 2 and perform activation of the data using the ReLU function after this step.

(g) Convolution layer 3: the result of batch normalization layer 2 is subject to two-dimensional convolution, with a 1 × 1 convolution filter, and the number of convolution kernels is 384.

(h) Batch normalization layer 3: after this step, the data in convolutional layer 3 are subjected to batch normalization and activation using the ReLU function.

(i) Fully connected layer 1: the number of nodes is 32, and all the data in batch normalization layer 3 are transformed into a one-dimensional format before being activated with the ReLU function.

(j) Fully connected layer 2: the number of nodes is three since the output of the CNN corresponds to three origins, i.e., Sichuan, Jiangsu, and Henan.

The parameters of the CNN must be determined through training, and a gradient descent method was employed to find the optimal network parameters. The cross-entropy loss was utilized to quantify the difference between predicted and actual results. The initial learning rate in this study was set to 0.0001, the mini-batch size was 4, and the maximum number of epochs was 300.

### 2.4. Performance Evaluation of Models

The model evaluation metrics adopted in this study included accuracy (*Acc*), precision (*P*), recall (*R*), F1 score (*F1*), and classification error (*E*). Accuracy was used to evaluate the model’s overall performance, while precision, recall, F1 score, and classification error were used to evaluate the model’s performance in each category. The calculation was as follows:

(6) $$Acc=\frac{TP+TN}{TP+TN+FP+FN}$$

(7) $$P=\frac{TP}{TP+FP}$$

(8) $$R=\frac{TP}{TP+FN}$$

(9) $$F1=\frac{2\times P\times R}{P+R}$$

(10) $$E=1-\frac{TP}{TP+FN}$$

where TP represents the number of positive samples that are classified as positive, *FN* represents the number of positive samples that are classified as negative, FP represents the number of negative samples that are classified as positive, and TN represents the number of negative samples that are classified as negative. For instance, when calculating Acc, P, R, F1, and E for duck eggs from Sichuan, samples from Sichuan are positive, whereas samples from Jiangsu and Henan are negative.

### 2.5. Online Nondestructive Detection Techniques and Devices

#### 2.5.1. Hardware of the Device

**Figure 3:**
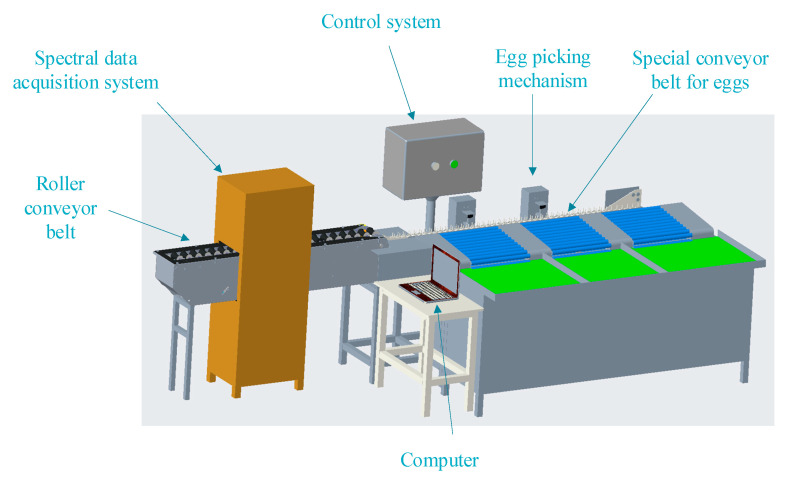
The device for detecting the origin of duck eggs.

The device mainly consists of a spectrum data acquisition system, a control system, a roller conveyor belt, an egg-picking mechanism, and a specific conveyor belt for eggs (Figure 3). A computer, a Maya2000Pro spectrometer, a black box, a light source, and an 84UV lens constitute the interior of the spectral data acquisition system, which has been presented in Figure 2. The roller conveyor belt’s function is to transport duck eggs. The control system consists of a lower computer, sensors, motors, and control switches. The lower computer is S7-200 PLC, and the sensor is CR-10P. In this study, the lower computer controls the egg-picking mechanism, starts and stops motor operation, and receives sensor signals. The sensors’ function is to send signals to the computer when the duck egg reaches a specified position, while the motor provides power for transportation. The special conveyor belt for eggs transports the duck eggs from a horizontal state to a vertical state.

#### 2.5.2. Software for Online Detection of Duck Egg Origin and Traceability

**Figure 4:**
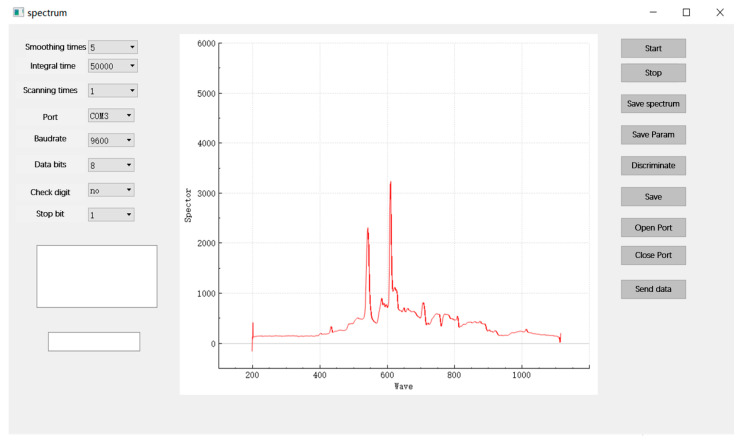
The software for online detection of duck egg origin and traceability.

The software for online detection of duck egg origin and traceability was developed based on Qt (Figure 4). The software’s main functions include spectrometer acquisition parameter setting, real-time display of spectral data, communication parameter setting with the lower computer, calling discriminative models, and other general operations such as spectral data saving. The core of the software is the calling of the spectrometer and discriminant model and the communication with the lower computer. The Ocean Optics OmniDriverSPAM-2.56 software development kit was utilized to call the Maya2000Pro spectrometer. The core of the OmniDriverSPAM development kit is the Wrapper, which provides APIs for configuring spectrum acquisition parameters such as spectrometer on/off, integration time setting, and scan count setting. There are two types of model calls: machine learning model calls and deep learning model calls, where machine learning models are called using C++ calls to Python scripts, and deep learning models are called using libtorch. BaudRate, DataBits, Parity, StopBits, and other connection parameters were configured using the QSerialPort class. This software was also used in Section 2.2 Spectral Data Acquisition System for data acquisition.

#### 2.5.3. Control System for Online Detection Devices

**Figure 5:**
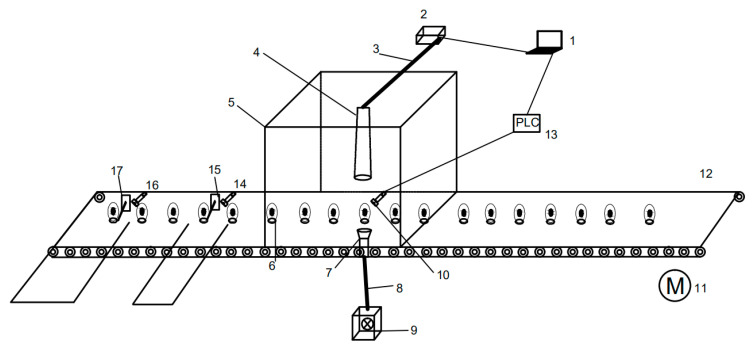
The schematic diagram of the workflow of online detection of duck egg origin and traceability. 1. Computer; 2. Spectrometer; 3. Data Cable; 4. 84UV lens; 5. Dark box; 6. Duck egg; 7. Focusing lens; 8. Glass optical Fiber; 9. Light source; 10. Sensor 1; 11. Motor; 12. Conveyor belt; 13. S7-200; 14. Sensor 2; 15. Egg-picking mechanism 1; 16. Sensor 3; 17. Egg-picking mechanism 2.

To develop a control system for the online detection of duck eggs, it is vital to comprehend the workflow of the online detection of duck eggs. The workflow is as follows (Figure 5): The duck egg is placed on the conveyor belt, and the motor drives the conveyor belt forward. When the duck egg reaches sensor 1, the sensor is triggered, and the conveyor belt stops for 0.5 s; the PLC receives the signal and transmits it to the spectral detection software; the spectral detection software collects the spectral data of the duck egg at this time, discriminates it immediately, and transmits the discrimination result to the PLC. When the duck egg reaches sensor 2, the PLC begins to transmit the discriminating signal to the egg-picking device; if the discrimination result is a duck egg of Sichuan origin, the egg-picking operation will be carried out; if the duck egg reaches sensor 3, the discrimination result is a duck egg of Jiangsu origin, and the egg-picking operation will be performed.

Once the device’s workflow is determined, the control system can be developed, mainly including the design of communication, input, and output, as well as the storage of duck egg discrimination results. The serial port was selected as the communication method, the device for online detection of duck egg origin and traceability had three input ports and three output ports, and the discrimination results were entered into registers in a first-in-first-out manner.

#### 2.5.4. Validation Experiments of Online Detection Devices

To test the detection performance of the device, 90 duck eggs (30 duck eggs from Sichuan, 30 from Jiangsu, and 30 from Henan) were marked and placed on the device for online detection of duck egg origin and traceability. Then, the device was launched, and the software was utilized to collect the spectral data of the duck eggs and determine their origins. Next, the PLC removed the duck eggs. Finally, the device’s discrimination results were counted, and the accuracy was calculated according to the results of marking and discriminating duck eggs.

## 3. Results and Discussion

### 3.1. Spectral Analysis of Duck Eggs

**Figure 6:**
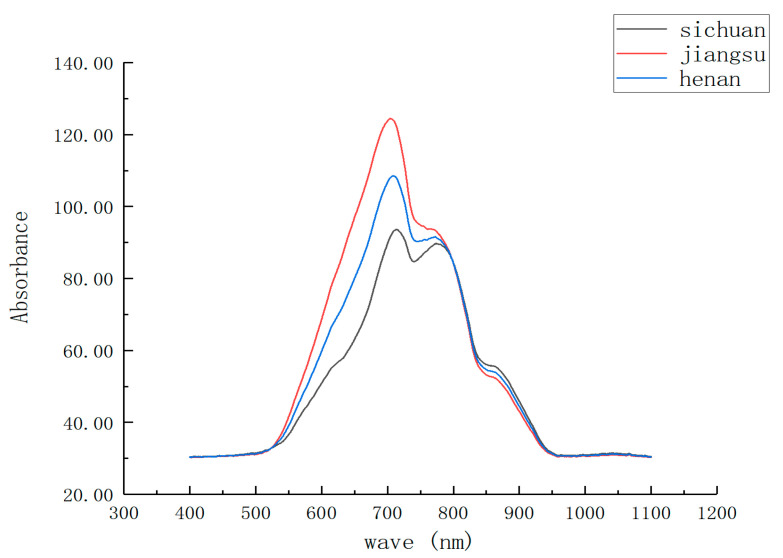
The average spectral value of duck eggs.

The raw spectra of duck eggs from Sichuan, Jiangsu, and Henan were averaged separately (Figure 6). It was found that the mean spectra of duck eggs from different origins differed significantly between 550 nm and 900 nm, which was a result of their different farming environments. Specifically, the average spectral value of the duck eggs from Jiangsu was the highest, that of the duck eggs from Sichuan was the lowest, and that of the duck eggs from Henan was in the middle. The absorption peaks at 700 nm and 800 nm in the spectra of duck eggs from the three origins were mostly related to the combined and multiplied absorption of water molecules, alcohol molecules, and O-H groups [34]. It was determined that the ratio of the two peaks was highest in Jiangsu, smallest in Sichuan, and intermediate in Henan. These discrepancies indicate that spectroscopy could be exploited to determine the origin of duck eggs.

### 3.2. Sensitive Band Analysis of Duck Eggs

**Figure 7:**
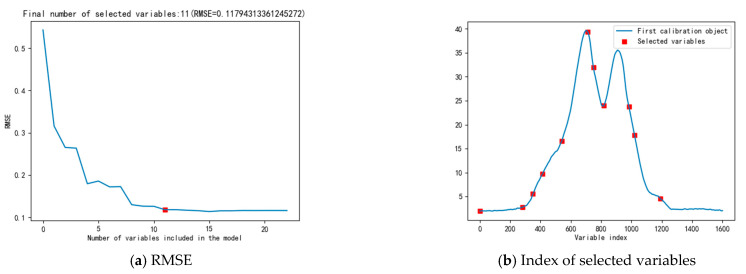
Sensitive bands selection based on SPA.

Before selecting the sensitive bands, SNV, SG, and MSC algorithms were used for pre-processing duck egg spectral data. Separately, the CARS and SPA algorithms were employed to extract the sensitive bands. Initially, SPA was used to extract the feature variables of the spectrum of duck eggs. According to the principle of the SPA algorithm, the transition point at which the derivative of the root mean square error (RMSE) becomes smaller is chosen through root mean square error (RMSE) minimization, and redundant information before the transition point is removed [35]. In Figure 7, the SG-preprocessed spectral data are presented as an example to explain how SPA extracts variables. Figure 7a shows the variation in RMSE with the selected variables, with the smallest RMSE value appearing when 11 variables were selected. Figure 7b shows the index of sensitive bands. Eleven characteristic wavelength points ranging from 400 to 930 nm were chosen. Following the pre-processing by SG, SNV, and MSC algorithms, Table 2 presents the distinctive wavelength points determined by SPA.

**Figure 8:**
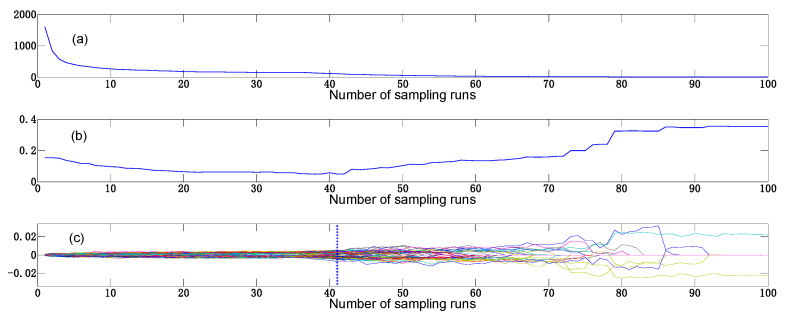
Sensitive band selection based on CARS. (**a**) The number of sampled variables. (**b**) RMSECV. (**c**) Regression coefficient path.

The CARS algorithm can reduce irrelevant variables from spectral data; the spectral data pre-processed by MSC is used to explain the process of variable extraction by CARS in Figure 8. The number of Monte Carlo samples was set to 100, and ten-fold cross-validation was used. As depicted in Figure 8a, the number of selected variables decreases gradually as the number of samples increases. The root mean square error of cross-validation (RMSECV) in Figure 8b decreased gradually from sampling 0 times to sampling 41 times, and the process eliminated a large amount of redundant information. However, the RMSECV value increased after sampling 41 times, indicating that the process eliminated useful information [36]. When the RMSECV value reached a minimum, the regression coefficients of each variable are shown at the vertical line in Figure 8c, and at this time, the optimal number of selected variables was 88, and the number of sample runs was 41. Following the pre-processing using the SNV and MSC algorithms, the number of selected variables was 100 and 132, respectively.

### 3.3. Classification Based on RF, SVM, and CNN

The selected variables of duck eggs were fed into the RF, SVM, and CNN models. Here, there were 10 decision trees for the RF model, and each tree had a depth of 2. The radial basis function (RBF) was employed as the kernel function of the SVM model. The classification results of the RF, SVM, and CNN models are listed in Table 3, Table 4 and Table 5, respectively. The RF model with the best performance was the one established after pre-processing with the SG algorithm and extracting feature wavelengths with the SPA algorithm. Specifically, the recall of duck eggs from Sichuan, Jiangsu, and Henan were 100.00%, 94.44%, and 96.55%, respectively. The precision of duck eggs from Sichuan, Jiangsu, and Henan were 100.00%, 94.44%, and 96.55%, respectively. The F1 scores of duck eggs from Sichuan, Jiangsu, and Henan were 100.00%, 94.44%, and 96.55%, respectively. The classification error of duck eggs from Sichuan, Jiangsu, and Henan were 0.00%, 5.56%, and 3.45%, respectively. The overall accuracy was 97.47%. 

For the SVM model, the performance based on the SNV algorithm and SPA algorithm was best. Specifically, the recall of duck eggs from Sichuan, Jiangsu, and Henan was 96.88%, 100.00%, and 100.00%, respectively. The precision of duck eggs from Sichuan, Jiangsu, and Henan was 100.00%, 94.74%, and 100.00%, respectively. The F1 score of duck eggs from Sichuan, Jiangsu, and Henan was 98.42%, 97.30%, and 100.00%, respectively. The classification error of duck eggs from Sichuan, Jiangsu, and Henan was 3.12%, 0.00%, and 0.00%, respectively. The overall accuracy was 98.73%. 

For the CNN model, the performance based on the SNV algorithm and SPA algorithm, MSC algorithm and SPA algorithm, SNV algorithm, CARS algorithm, and MSC algorithm and CARS algorithm was the best. Specifically, the recall of duck eggs from Sichuan, Jiangsu, and Henan was 100.00%, 100.00%, and 100.00%, respectively. The precision of duck eggs from Sichuan, Jiangsu, and Henan was 100.00%, 100.00%, and 100.00%, respectively. The F1 score of duck eggs from Sichuan, Jiangsu, and Henan was 100.00%, 100.00%, and 100.00%, respectively. The classification error of duck eggs from Sichuan, Jiangsu, and Henan was 0.00%, 0.00%, and 0.00%, respectively. The overall accuracy was 100.00%. 

### 3.4. Selection of Device Models for Online Detection

The goal of developing RF, SVM, and CNN models is to apply them to the device; the model is desired to run faster and have high detection precision. The number of input variables and the size of the model determines the model’s running speed. The three models built by pre-processing with the MSC algorithm and extracting feature variables with the CARS algorithm are taken as an example to illustrate their size. As shown in Table 6, the size of the RF, SVM, and CNN models was 14 KB, 208 KB, and 816 KB, respectively. The CARS algorithm extracts a larger number of feature variables from the spectra of duck eggs than the SPA algorithm (as shown in Table 3, Table 4 and Table 5). Considering the accuracy of the models, the CNN model has the highest detection accuracy at 100.00%, followed by the SVM model at 98.73% and the RF model at 97.44%. For use on the device, the following were selected: the RF model based on the duck egg spectra, pre-processed by the SG and SPA algorithms; the SVM model based on the duck egg spectra pre-processed by the SNV and SPA algorithms, and the CNN model based on the duck egg spectra pre-processed by the SNV and SPA algorithms. 

### 3.5. Performance of the Detection Device

After determining the pre-processing algorithm, the feature variable extraction algorithm, as well as the model to be applied on the device, with a detection performance of 90 duck eggs was tested on the device. Using the software for online detection of duck egg origin and traceability, the spectral data of the duck eggs were collected, and the characteristic wavelength points were extracted. Then, the model was used to discriminate the origin of the duck eggs, and finally, the duck eggs of different origins were sorted by the device based on the discriminated results. The results are shown in Table 7. It can be seen that the RF model based on the duck egg spectra pre-processed by the SG and SPA algorithms obtained the worst performance. The overall accuracy rate was 90.00%. For the SVM model, the SNV algorithm was used to pre-process the duck egg data, and the SPA algorithm was used to extract the feature variables. The overall accuracy rate was 91.11%. The CNN model based on the duck egg spectra pre-processed by the SNV algorithm and the SPA algorithm achieved the best performance. The overall accuracy rate was 94.44%. The detection speed of the RF, SVM, and CNN models was 0.1 s/egg, 0.3 s/egg, and 0.5 s/egg, respectively.

### 3.6. Discussion

This study collected visible/near-infrared spectral data of duck eggs and developed classification models based on RF, SVM, and CNN for identifying duck egg origin. Based on this, a device was developed for the online detection of duck egg origin. This technology fills in the blanks in the detection of duck egg origin. Rock et al. employed the stable isotope technique for egg traceability and detection, which requires breaking the eggshell, sterilizing the egg, and measuring its C, N, O, and S isotopic composition. Furthermore, the operation is complicated, and the detection speed is slow; it requires a minimum of 2 days to test each sample [4]. Compared to the stable isotope technique, the visible/near-infrared spectral technique utilized in this study provides a substantially faster detection speed and does not require breaking the duck eggshell. Sun et al. collected images of eggs using a hyperspectral camera with a spectral range of 871.607~1766.322 nm using the CS-SVM approach to establish an origin discrimination model with a model accuracy of 99.3% [5]. Yet, the hyperspectral methodology is expensive, and the experimental method cannot be applied to real production. Moreover, the NMR technology has disadvantages such as expensive price and slow speed; each sample required 9.1 seconds to acquire the NMR data [6]. Liu et al. utilized a Fourier transform near-infrared (FT-NIR) spectrometer to collect spectral data on duck eggs. Applying machine learning techniques, the classification accuracy for distinguishing eggs of different origins was found to be 93.8%. The detection speed of the FT-NIR spectrometer remains a potential limitation in practical applications [7]. The method and technique described in this study are precise, economical, fast, and directly applicable to production practice. Notably, this study extends beyond previous works which focused exclusively on chicken eggs.

In addition, there are numerous instances of combining CNN with spectroscopic techniques to detect agricultural products. For example, Yu et al. utilized a one-dimensional CNN (1D-CNN) to predict the pesticide residues in Hami melon [31]. Tian et al. used a 1D-CNN combined with visible/near-infrared spectroscopy to achieve the detection of freezing damage in oranges [32]. Unfortunately, these are only theoretical and algorithmic research. Our study combines CNN with spectroscopic techniques for practical production.

## 4. Conclusions

This paper proposed a technique and method for the origin and traceability detection of duck eggs based on visible/ near-infrared spectroscopy, with a particular application to the online detection of duck egg origin. The SPA and CARS algorithms were used to extract feature variables from the spectra of duck eggs. RF, SVM, and CNN models were established, and online validation experiments were conducted. The experimental results indicated that the SPA algorithm was more suitable for feature variable extraction for detecting the origin of duck eggs, and the accuracy of the established RF, SVM, and CNN models was 97.47%, 98.73%, and 100%, respectively. When applied to the device, the RF, SVM, and CNN models achieved an accuracy of 90%, 91.1%, and 94.44%, respectively. The results verify that the visible near-infrared spectroscopy technology could be exploited to achieve online detection of the origin of duck eggs, providing an immediately applicable technical solution for the duck egg production industry to ensure the quality of duck eggs and consumer rights.

## Figures and Tables

**Table 1:** The detailed distribution of the duck egg data.

	Jiangsu	Sichuan	Henan	Total
Training set	70	56	56	182
Test set	30	24	25	79
Online detection	30	30	30	90
Total	130	110	111	351

**Table 2:** The characteristic wavelength points selected by SPA.

Pre-Processing	Wavelength (nm)
SG	400; 527.67; 559.26; 587.59; 644.38; 719.71; 736.44; 766.27; 838.55; 854.55; 927.96
SNV	439.45; 522.24; 557.01; 583.55; 610.9; 710.45; 722.79; 1075.42
MSC	440.82; 459.54; 517.71; 522.69;534.45; 563.77; 587.14; 610.9; 717.06; 732.04; 957.33; 1053.34; 1068.76

**Table 3:** The results of the RF model.

Pre-Processing	VN	Results						
		Category	Number	P/%	R/%	F1/%	E/%	Acc/%
SG + SPA	11	0	32	100.00	100.00	100.00	0.00	97.47
		1	18	94.44	94.44	94.44	5.56	
		2	29	96.55	96.55	96.55	3.45	
SNV + SPA	8	0	32	100.00	96.88	98.42	3.12	94.94
		1	18	85.00	94.44	89.47	5.56	
		2	29	96.43	93.10	94.74	6.90	
MSC + SPA	13	0	32	88.24	93.75	90.91	6.25	89.87
		1	18	78.95	83.33	81.08	16.67	
		2	29	100.00	89.67	94.55	10.33	
SG + CARS	88	0	32	100.00	100.00	100.00	0.00	93.67
		1	18	78.26	100.00	87.80	0.00	
		2	29	100.00	82.78	90.58	17.22	
SNV + CARS	100	0	32	100.00	100.00	100.00	0.00	93.67
		1	18	78.26	100.00	87.80	0.00	
		2	29	100.00	82.76	90.57	17.24	
MSC + CARS	132	0	32	100.00	100.00	100.00	0.00	94.94
		1	18	85.00	94.44	89.47	5.56	
		2	29	96.30	89.66	92.86	10.34	

Category: 0, duck eggs from Sichuan; 1, duck eggs from Jiangsu; 2, duck eggs from Henan. VN: number of variables.

**Table 4:** The results of the SVM model.

Pre-Processing	VN	Results						
		Category	Number	P/%	R/%	F1/%	E/%	Acc/%
SG + SPA	11	0	32	100.00	96.88	98.42	3.12	92.41
		1	18	75.00	100.00	85.71	0.00	
		2	29	100.00	82.76	90.57	17.24	
SNV + SPA	8	0	32	100.00	96.88	98.42	3.12	98.73
		1	18	94.74	100.00	97.30	0.00	
		2	29	100.00	100.00	100.00	0.00	
MSC + SPA	13	0	32	100.00	87.50	93.33	12.50	93.67
		1	18	78.26	100.00	87.80	0.00	
		2	29	100.00	96.66	98.30	3.34	
SG + CARS	88	0	32	100.00	87.50	93.33	12.50	78.48
		1	18	51.42	100.00	67.92	0.00	
		2	29	100.00	55.17	71.11	44.83	
SNV + CARS	100	0	32	100.00	100.00	100.00	0.00	96.20
		1	18	85.71	100.00	92.31	0.00	
		2	29	100.00	89.66	94.55	10.34	
MSC + CARS	132	0	32	100.00	100.00	100.00	0.00	94.94
		1	18	81.82	100.00	90.00	0.00	
		2	29	100.00	86.21	92.59	13.79	

Category: 0, duck eggs from Sichuan; 1, duck eggs from Jiangsu; 2, duck eggs from Henan. VN: number of variables.

**Table 5:** The results of the CNN model.

Pre-Processing	VN	Results						
		Category	Number	P/%	R/%	F1/%	E/%	Acc/%
SG + SPA	11	0	32	100.00	100.00	100.00	0.00	98.73
		1	18	94.74	100.00	97.30	0.00	
		2	29	100.00	96.55	98.24	3.45	
SNV + SPA	8	0	32	100.00	100.00	100.00	0.00	100.00
		1	18	100.00	100.00	100.00	0.00	
		2	29	100.00	100.00	100.00	0.00	
MSC + SPA	13	0	32	100.00	100.00	100.00	0.00	100.00
		1	18	100.00	100.00	100.00	0.00	
		2	29	100.00	100.00	100.00	0.00	
SG + CARS	88	0	32	100.00	100.00	100.00	0.00	98.73
		1	18	100.00	94.44	97.14	5.56	
		2	29	96.67	100.00	98.31	0.00	
SNV + CARS	100	0	32	100.00	100.00	100.00	0.00	100.00
		1	18	100.00	100.00	100.00	0.00	
		2	29	100.00	100.00	100.00	0.00	
MSC + CARS	132	0	32	100.00	100.00	100.00	0.00	100.00
		1	18	100.00	100.00	100.00	0.00	
		2	29	100.00	100.00	100.00	0.00	

Category: 0, duck eggs from Sichuan; 1, duck eggs from Jiangsu; 2, duck eggs from Henan. VN: number of variables.

**Table 6:** The size of the models.

Model	Size (KB)
RF	14
SVM	208
CNN	816

**Table 7:** The test results of the device.

Model	VN	Results							
		Category	Number	P/%	R/%	F1/%	E/%	Acc/%	Speed/s/egg
RF	11	0	30	87.10	90.00	88.53	10.00	90.00	0.1
		1	30	100.00	80.00	88.89	20.00		
		2	30	85.71	100.00	92.31	0.00		
SVM	8	0	30	90.63	96.67	93.55	3.33	91.11	0.3
		1	30	89.66	86.67	88.14	13.33		
		2	30	93.10	90.00	91.52	10.00		
CNN	8	0	30	93.55	96.67	95.08	3.33	94.44	0.5
		1	30	96.55	93.33	94.91	6.67		
		2	30	93.33	93.33	93.33	6.67		

Category: 0, duck eggs from Sichuan; 1, duck eggs from Jiangsu; 2, duck eggs from Henan. VN: number of variables.

## Data Availability

Our research data will need to be further used in future studies. Data sharing is not applicable to this article.
